# Deep Convolution Neural Network for Malignancy Detection and Classification in Microscopic Uterine Cervix Cell Images

**DOI:** 10.31557/APJCP.2019.20.11.3447

**Published:** 2019

**Authors:** Shanthi P B, Faraz Faruqi, Hareesha K S, Ranjini Kudva

**Affiliations:** 1 *Department of Computer Science and Engineering, *; 2 *Department of Computer Applications, Manipal Institute of Technology, *; 3 *Department of Pathology, Kasturba Medical College, Manipal Academy of Higher Educaton, Udupi, Karnataka, India. *

**Keywords:** Pap smear, cervical screening, cell image classification, convolution neural network, deep learning

## Abstract

**Objective:**

Automated Pap smear cervical screening is one of the most effective imaging based cancer detection tools used for categorizing cervical cell images as normal and abnormal. Traditional classification methods depend on hand-engineered features and show limitations in large, diverse datasets. Effective feature extraction requires an efficient image preprocessing and segmentation, which remains prominent challenge in the field of Pathology. In this paper, a deep learning concept is used for cell image classification in large datasets.

**Methods:**

This relatively proposed novel method, combines abstract and complicated representations of data acquired in a hierarchical architecture. Convolution Neural Network (CNN) learns meaningful kernels that simulate the extraction of visual features such as edges, size, shape and colors in image classification. A deep prediction model is built using such a CNN network to classify the various grades of cancer: normal, mild, moderate, severe and carcinoma. It is an effective computational model which uses multiple processing layers to learn complex features. A large dataset is prepared for this study by systematically augmenting the images in Herlev dataset.

**Result:**

Among the three sets considered for the study, the first set of single cell enhanced original images achieved an accuracy of 94.1% for 5 class, 96.2% for 4 class, 94.8% for 3 class and 95.7% for 2 class problems. The second set includes contour extracted images showed an accuracy of 92.14%, 92.9%, 94.7% and 89.9% for 5, 4, 3 and 2 class problems. The third set of binary images showed 85.07% for 5 class, 84% for 4 class, 92.07% for 3 class and highest accuracy of 99.97% for 2 class problems.

**Conclusion:**

The experimental results of the proposed model showed an effective classification of different grades of cancer in cervical cell images, exhibiting the extensive potential of deep learning in Pap smear cell image classification.

## Introduction

Cancer of the uterine cervix is one of the most common gynecological cancers and is the leading cause of mortality and morbidity among women worldwide. A malignant tumor occurs when the cervix cells grow and replicate abnormally with uncontrolled cell division and cell death. According to the recent report received from Information Centre on HPV and Cancer ICO/ IARC, cervical cancer is the fourth most common and frequent cancer among women. Recent estimates of ICO/IARC, indicate that every year 527,624 women are diagnosed with cervical cancer, and 265,672 die from cancer. This is due to poor access to screening and treatment services especially for women living in low and middle-income countries (Bruni et al., 2015). Cervical cancer is a slow growing cancer that takes nearly 10 to 20 years to show its symptoms. Therefore, routine usage of Pap smear test in developing and underdeveloped countries helps in reducing mortality and morbidity rate of this cancer. It is completely preventable and curable, if detected and treated the pre-cancerous symptoms at the early stages (Takiar et al., 2010). Thus, automating the process helps the pathologists to analyze a large number of samples in a short period. The epithelium that covers the cervix is known as the squamous epithelium, and over 90% of cancer occurs in such squamous cells. According to WHO classification, the initial and mild stage of cancer is termed as mild dysplasia, which later advances to the next stage called moderate dysplasia, followed by severe dysplasia and carcinoma, and finally to invasive cancer that invades to other parts of the body. In Cervical Intraepithelial Neoplasia (CIN) system, mild dysplasia is classified as CIN1, moderate dysplasia as CIN2, severe and carcinoma as CIN3 and the final stage as invasive cancer. 

Bethesda system is a standard system followed worldwide in reporting the cases in diagnosing cervical cancer. This system further eliminates the sub classification of CIN by classifying CIN1 as Low-Grade Intraepithelial Lesion (LSIL) and CIN2 and CIN3 as High-Grade Intraepithelial Lesion (HSIL) and the last stage as invasive cancer (Reynolds et al., 2004; Ehrmann, 1996). A tremendous advancement in image acquisition devices aided in acquiring a large amount of data that initiated a big challenge for image analysis. Traditional machine learning techniques have helped massively in automating the diagnosis process, but they failed in dealing with large volume of data. 

Deep learning algorithms (LeCun et al., 2015) and architectures mitigate the issues on big volume of data. The objective of this learning process is to learn a complex and abstract representation of data in a hierarchical manner by passing the data through multiple transformation layers. The convolution neural network (CNN) (Krizhevsky et al., 2012) is one such framework that performs exceptionally well for high dimensional data, as it learns the underlying complex function empirically, and shows better performance than the traditional machine learning algorithms. Hence, the CNN is used for classifying the cervical cells in cytological images by giving whole single cell image as input instead of using manually extracted features of nucleus and cytoplasm of the cervical cell images. The traditional machine learning methods include cell segmentation, feature extraction/selection and classification to classify the images. The successful classification techniques among traditional methods rely on the accuracy of the segmented features. Effective cell nucleus segmentation is a crucial task when the severity of the disease moves from moderate to severe and to carcinoma, as it is extremely essential for accurate feature extraction. After optimizing the cytoplasmic and nucleus features, 96.8% is the highest accuracy achieved on the Herlev dataset (Marinakis et al., 2009). In the prior work of authors (Shanthi and Hareesha, 2019), the cytoplasmic features were excluded and attained high classification accuracy by using only the nucleus and its textural features for classification. However, this model’s performance decreased when applied to larger datasets. Instead, convolution, a key component of a CNN network scales up effectively on high dimensional data and plays an important role in the success of image processing. Due to this property, these models pose a great impact in the field of health informatics (LeCun et al., 2015; Shin et al., 2016; Lee et al., 2017). 

In this paper, the CNN is used for classifying the cervical cells of cytological images. This deep neural network convolves the learned features from the input images and uses 2D convolution kernels to extract abstract and complex features. The CNN eliminates manual feature extraction by making the network to learn meaningful features from the input data repeatedly, when they train on a collection of labeled images. This automated feature extraction makes the deep learning model highly accurate for image analysis task, but also requires a large dataset to work. We used a new Pap smear database developed by Herlev University Hospital for this work. The single cervical cell images of Herlev Dataset were collected using a digital camera and microscope under a resolution of 0.201 µm/pixel (Jantzen and Dounias, 2006; Marinakis et al., 2009; Lee et al., 2017). The Pap smear database consists of 917 images distributed unequally on seven different classes. The proposed study includes 749 images with five different grades of disease like carcinoma, severe, moderate, mild and normal. To increase the volume of the dataset, Pap smear images used for the study were augmented to 10,000 images on each grade and thereby expanded the volume of dataset to 50,000 images.

The CNN needs a large amount of training data to achieve good performance. Image augmentation (Lee et al., 2017; Taha et al., 2017 ; Zhang et al., 2017) helps in creating more number of training images artificially through different ways of processing such as random rotation, translation, scaling and flipping. In cervical cell images, size and intensity of the nucleus are considered the key features to distinguish the normal and the abnormal cells. Even though multiple data instances have been created from the same image, each image has a unique pixel distribution due to randomized sampling from multiple methods. This results in the creation of multiple training data instances from the same image. Data augmentation process helps in generating an adequate amount of data that aids the CNN to learn meaningful features and it results in good accuracy and convergence rate in image classification.

*Related Work*

Early cancer diagnostics is the crucial task for health care as the tumor detection period is tightly bound to survival rates. Artificial neural network, inspired by human biological nervous system is used to solve a complex pattern that includes layered architecture with one or more hidden layers (Lee et al., 2017). Deep learning is the enhancement in the artificial neural network that consist of hundreds of hidden layers and captures non-linear relationship of the complex pattern and makes intelligent predictions. The convolution neural network (CNN) is one of the most popular deep neural network architectures that have useful application in image recognition and analysis (Zhang et al., 2017). It automatically learns feature at multiple levels of abstraction that allows a system to learn all possible features from low-level features such as edges and pixel intensity of an image to high-level features such as objects and shapes without depending on human extracted features (Zhang et al., 2017).

The success of the traditional classification method mainly depends on the accuracy of the cell segmentation to extract features. Taha et al., (2017) proposed an idea to classify the cells directly without prior segmentation based on the deep feature learning using convolution neural network. They achieved good classification result and acquired 98.3 % accuracy on Herlev data set. Zhang et al., (2017), presented a deep learning solution for cervical cancer screening in which pre-trained CNN architecture is used to extract the features and these features are given as input to SVM classifier and attained good classification results on Herlev dataset. The article (Ravì et al., 2017) focuses on key applications in deep learning especially in the area of bio-informatics, medical imaging, medical informatics, and public health. The authors compared different architectures of deep learning and outlined the advantage of deep learning in health informatics by allowing automatic generation of features which reduces the amount of human involvement in the feature extraction process. Shin et al., (2016), evaluated the CNN performance on thoracic-abdomen lymph node detection and interstitial classification of lung disease. Based on the empirical evaluation and performance analysis, the CNN is suggested for high performance computer-aided detection for other medical imaging tasks. Automated tissue characterization is one of the crucial tasks of computer aided diagnosis for interstitial lung diseases. Anthimopoulos et al., (2016) proposed deep learning techniques using CNN for the classification of interstitial lung disease and achieved 85.5% classification performance in analyzing the lung patterns.

Hyeon et al., (2017) trained a model automatically to classify normal and abnormal state of cervical cells from microscopic images using a CNN and extracted feature vectors of cervical cell images. These extracted features were trained using SVM classifier and acquired a performance of 78%. Based on the deep learning algorithms, the prostate cancer classification model is proposed in this study and achieved an accuracy of 80.1% and 78.1% on training and testing sets. Huang (2017) presented a CNN based transfer learning to classify an image of diabetic retinopathy fundus. A pre-trained CNN model is used to extract features from fundus images, and the SVM is trained using the features and have shown better classification results. Devi et al., (2016) analyzed different type of architecture in ANN such as multi-layered perceptron, back propagation neural network, radial basis function network, fuzzy RBF network, convolution neural network, and feed forward network. The study revealed that the performance of ANN could be enhanced by embedding the learning capability which helps to increase its efficiency with the evaluation of good performance.

Mundhra et al., (2017) made a study on automated peripheral blood smear analysis system and employed deep learning models to analyze peripheral blood smear for localization and classification. The authors achieved 98% and 91% of specificity and sensitivity. Another study by Sornapudi et al., (2018) used deep learning based nuclei segmentation approach on gathering localized information through the generation of super pixel using a simple iterative clustering algorithm and training with the convolution neural network. The proposed approach achieved an overall nuclei detection accuracy of 95.97%.

## Materials and Methods

This section explains the method adopted to classify the cervical cell images of Herlev dataset using the Convolution Neural Network. The study included 749 images from Herlev Pap smear dataset, used five different data preprocessing algorithms to enhance the images and adopted two validation procedures to select the best preprocessing algorithm among five different algorithms used for image enhancement. As the part of first validation procedure, enhanced images were shown to experts in Pathology department of Kasturba Medical College, Manipal, India and their inputs were used for further analysis of images. Secondly, nucleus portion of the cell images were segmented and compared with benchmark nucleus images and the preprocessing method with good segmented accuracy (Shanthi and Hareesha, 2019) was taken for further study. Since the deep learning networks need a large amount of training data to achieve good performance, the data were augmented artificially creating multiple unique training samples from the same image. Finally, the proposed convolution neural network model is used to extract complex features from cervical cell image automatically, and classified different stages of cervical cancer such as normal, mild, moderate, severe and carcinoma.

*Dataset*

Pap smear data set is a collection of cervical single cell images of uterine cervix. The cervical cell images used for this classification study using the deep learning were obtained from Herlev dataset which is publicly available. The images were collected from Herlev University Hospital, Denmark employing digital camera and microscope with a resolution of 0.02 µm / pixel by the skilled cyto-technicians and doctors (Jantzen and Dounias, 2006). Pap smear database consists of 917 single cell samples distributed on seven different classes shown in Table 1. The study considered five classes out of seven that include normal, mild, moderate, severe and carcinoma for classification. For analysis purpose, 749 images were used from the Herlev dataset comprising 74 cells of normal superficial, 182 cells of mild dysplasia, 146 cells of moderate dysplasia, 197 cells of severe dysplasia and 150 cells of squamous cell carcinoma.

*Data Preprocessing*

**Figure 1:**
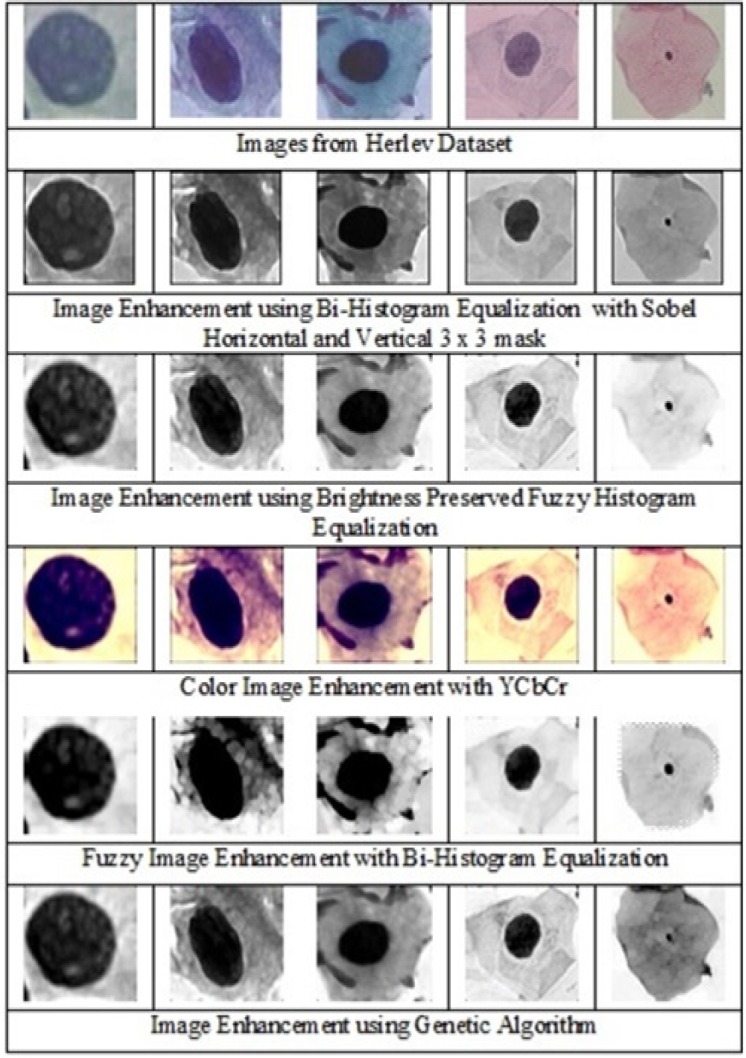
Enhanced Cervical Single Cell Images of Carcinoma, Severe, Moderate, Mild and Normal Grades Using Various Image Enhancement Techniques

The goal of data preprocessing is to augment the important features for subsequent image analysis. For this, it is essential to define the features that are important for the task. In cervical cells, the nucleus is one of the most prominent biomarker for disease diagnosis. In normal cells, the size of the nucleus will be tiny with the dark smooth texture. When the severity of the disease increases, the size of the nucleus slowly distorts. Based on the cell nucleus size, the cervical cells are graded as normal, mild as CIN1, moderate as CIN2, severe and carcinoma as CIN3. Five different image enhancement algorithms were used for enhancing the 749 cell images of normal, mild, moderate, severe and carcinoma, and the enhanced output images of 5 different grades are shown in Figure 1.

Five different image enhancement algorithms were used to find the perfect fit empirically. Bi-Histogram Equalization with adaptive sigmoidal function combined with sobel operator (horizontal and vertical), is used to highlight the edges and reduce the over-enhancement and brightness caused by Histogram Equalization (Arriaga-Garcia et al., 2014). Brightness-preserved Dynamic Fuzzy Histogram equalization is used to maintain mean brightness and preserve the image appearance (Ibrahim and Kong, 2007). Color image enhancement using YCbCr color space is used for presenting a better view of cell images (Srinivasan et al., 2005). Fuzzy image enhancement using Bi-Histogram Equalization is used for enhancing the cervical cell images (Li et al., 2011). Fuzzy Image Mapping increases the ability to judge the level of edge pixels and improves the quality of the image. The image enhancement using genetic algorithm combined with Bi-Histogram Equalization (Hashemi et al., 2010) is used to enhance the cell images. In this simple chromosome structure is defined and it is used to find the best gray level to enhance the images.

As the part of validation procedure, we showed the enhanced images to the experts in the department of Pathology, Kasturba Medical College, Manipal, India and collected the inputs using the questionnaire comprising the options of grading the appearance of the images as very good, good, fair and poor. In this, Bi-Histogram Equalization with adaptive sigmoidal function combined with sobel operator (horizontal and vertical) algorithm showed good accuracy among all other algorithms. Secondly, nucleus portion of the cervical cell images were segmented from each method of the enhanced images and matched with the benchmark nucleus image of Herlev dataset and calculated the accuracy (Shanthi and Hareesha, 2019). Bi-Histogram Equalization (Arriaga-Garcia et al., 2014) with adaptive sigmoid function method combined with sobel horizontal, vertical mask showed good accuracy among all preprocessing methods. Therefore, this method was incorporated in enhancing the images as it enhanced the features in the best manner with good results. The cervical cell images of different grades such as normal, mild, moderate, severe and carcinoma were enhanced using the validated preprocessing method, Bi-Histogram Equalization (Arriaga- Garcia et al., 2014) with adaptive sigmoid function method combined with sobel horizontal, vertical mask that avoids over enhancement of the image and performs smooth transitions on gray tones and enhances the image more precisely preserving the mean brightness. 

*Data Augmentation*

Deep neural networks require a large amount of training data to achieve good performance. If the original data set has limited number of training samples, the data can be augmented by various means of processing such as scaling, translation, rotation at fine angles and flipping. With data augmentation, the adequate amount of images can be produced with variations so that the network can learn meaningful features from the image dataset. As explained previously, even though multiple data instances have been created from the same image, each image has a unique pixel distribution leading to the creation of multiple training data instances from the same image. Size and intensity of the nucleus are the important features used in classifying the images as normal and abnormal. In Herlev data set, as the abnormal cells are more than the normal cells, disease diagnosis will be a bias towards the abnormal cells in the classifier. This has to be balanced to improve the performance of the classifier and improve the accuracy and the convergence rate of CNN (Taha et al., 2017; Ker et al., 2018). To exclude any bias in the classification, equal amounts of instances of each class were maintained in the training process. Many experiments were performed using different factors and parameters to ascertain the maximum amount of augmentation without replication and executed using the library Keras (Chollet, 2015), which allowed us to try out many different combinations of parameters and fine tune the augmentation process. The final values used for augmenting the dataset is provided in Table 2. These values are optimal and any increase in these values will not be a unique addition to the number of training samples generated or diminish the features in the images.

**Figure 2:**
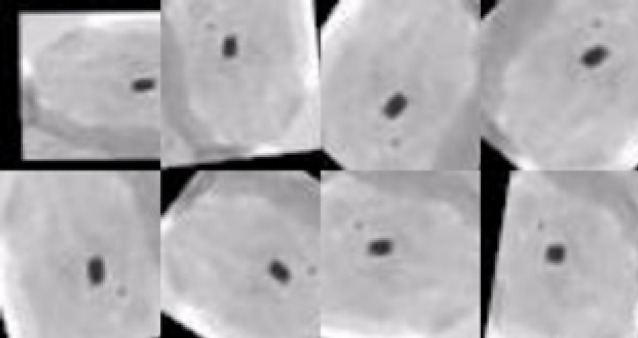
Different Orientation of Augmented Images Generated From a Single Cervical Cell Image of Mild Grade

A width shift range of 0.2 signifies that the image was shifted by a factor of 0.2 on both sides. This did not have a major effect on the image features and looked almost the same to the human eye. However, due to the displacement of the pixels in the image, it produced a unique training sample for the neural network and similar changes were made in the training samples using other factors. Use of multiple compound parameters helped us to generate a huge amount of training samples from a relatively small image set. Using this method, increased the total size of dataset from 300 to 10,000 for each class. The dimensions of each image are 50x50 pixels. Figure 2 shows the effect of augmentation process on one set of images. All eight of these training samples were extracted from the same image. Using this augmented training data, created a rich dataset to train the CNN model. The proposed CNN model were trained using balanced data inputs, with an equal number of samples for all the classes. The dataset was split as 80%, 10% and 10% for training, validation and testing purposes respectively. For the five class classification, 40,000 images were used to train the model, containing 8,000 samples from each of the classes. After training, 5,000 unique images were used for each of the validation and testing phases. Similarly, balanced splits were made for the different number of classes.

*Convolution Neural Network Architecture*

Convolution neural network (Krizhevsky et al., 2012) is one of the most popular machine learning algorithms widely used in image recognition and visual learning tasks. It has the greatest impact in the field of health informatics and medical image analysis domain (Mundhra et al., 2017; Suzuki 2017). One of the unique characteristics of this deep neural network is preserving the local image relation while performing the dimensionality reduction. This helps in capturing an important feature relationship in an image and reduces the number of parameters to compute the process thereby increasing the computational efficiency (Ibrahim and Kong, 2007; Ker et al., 2018). It is an example of supervised machine learning algorithms which require a significant amount of training data for proper learning and good classification. Its architecture can be defined as an interleaved set of feed forward layers implementing convolution filters followed by reduction, rectification or pooling layers which down-samples the feature map by summarizing the feature responses. The CNN architecture consists of sequences of convolution and sub sample layers. After the final sub-sampling layer, the CNN adopts several fully connected layers, which convert 2D feature maps to 1D vector that results in the final classification (LeCun et al., 2015; Kraus et al., 2016; Suzuki, 2017; Ker et al., 2018).

*Experimental Setup and Results*

**Figure 3:**
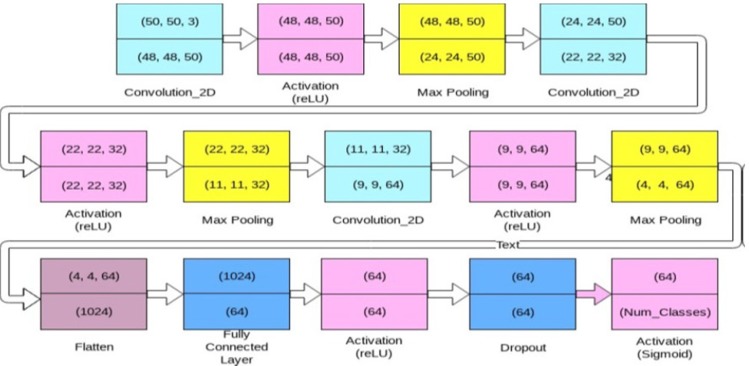
Architecture of Convolutional Neural Network Model Used for the Classification of Cervical Cell Images

The authors performed a set of experiments to gauge the classification abilities of a convolution network in classifying the cancerous images. Different taxonomies of cells, as given in the literature, were used to study and classify the images. These taxonomies give an insight into how to improve the classification accuracies by using better definitions of the classes while performing these experiments. The proposed model which is shown in Figure 3, explains the analysis of the method used for defining the classes. In all the different experiments, the data was split into three sets: training, validation, and testing. The training set contained 80% of the images, while the validation and test set contained 10% each. When combining different classes of images, or using different taxonomies, a balance was maintained in the relative sizes of the different classes for accurate results. Each class had the same amount of contribution (regarding the number of images) towards training, validation and test set, across all the classification experiments.

*Implementation*

The authors experimented with several different configurations to find the best fit model to model the CNN network. Figure 3 shows the model which gave the highest accuracy. This model has three 2D convolutional layers, each followed by an activation layer and a max pooling layer. The first layer of ConvNet has a size 50x50x3 (W1xH1xD1), where W1, H1 and D1 are 50 pixel width, 50 pixel height and 3 represents the depth of the image. The depth of the grayscale and binary images used in the study were made to three channels before inputting into the convolutional layer. The first convolutional layer has 50 filters with a kernel of size 3x3. Similarly, the second and third convolutional layers have 32 and 64 filters respectively, each of size 3x3. We used a stride (S) of 1 for each filter and spatial extent (F) as 3 and no zero padding (P=0). W1, H1 and D1 are resized to W2, H2 and D2 based on the four hyper parameters such as filters (K), spatial extent (F), stride (S) and zero padding (P) and calculated as:

W 2 = (W 1 − F + 2P )/S + 1                              (1)

H2 = (H1 − F + 2P )/S + 1                              (2)

D2 = K                              (3)

The equations (1), (2) and (3) produces a new volume of sizes for 2nd and 3rd convolutional layers with parameters F=3 (spatial extent of filter), P=0 (no zero padding used) and stride S = 1. Rectified Linear Unit (reLU), as presented in Equation (4), is an activation function used for faster and effective training of deep neural networks on large and complex datasets. Goodfellow (2013) used reLU (Rectified Linear Unit) as the activation function which performs non-linear transformation to the input and make the model to learn and perform more complex tasks and produces the best results empirically.

f (x) = max(0, x)                               (4)

After each activation, the max pooling was performed to down sample the features while retaining those higher values, i.e., signified important features. A 2- dimensional max pooling layer of size 2x2 downscales the features in both the spatial dimensions by half. At the end of the three convolutions, the resulting matrix is flattened, and passed through two densely connected layers, separated by a reLU activation layer. It was empirically found that using a dropout (Srivastava et al., 2014) performance of the model’s ability increases to generalize over newer samples, and escalates the test accuracy. The best results were obtained by keeping the value of dropout at 0.3. Finally, we added a sigmoid layer shown in Equation (5) to convert the values into probabilities. The batch size was kept at 128 which is the number of samples that are passed to the network at once.

$\left(z\right)=\frac{1}{1+e^{-z}}$                              (5)

We used the argmax function which outputs large as possible to find the best possible class for a particular test image and used multi-class Binary Cross Entropy (Mannor et al., 2003) as our loss function and RMSProp (Chollet, 2015) as our optimizer for training this model.

$-\sum_{=1}^{M}y_{\mathrm{o.c}}\log(p_{o,c})$                              (6)

The loss function has been presented in Equation (6). Here, M is the number of classes, y is the binary indicator (0 or 1), class label c is the correct classification for observation o, and p is predicted probability observation.

*Taxonomy of Cancer Cells*

**Figure 4:**
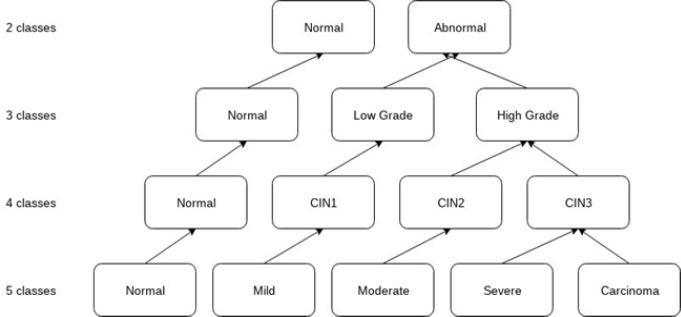
Different Taxonomies of Cervical Cell Images Employed on 3 Different Sets for Cell Image Classification

To appreciate the hidden complexity better in classifying cancerous images, we used different taxonomies, as specified in the literature. The first method of classification, used five classes namely Normal, Mild, Moderate, Severe and Carcinoma. Each of these classes contained 10,000 images, obtained using the procedure of data augmentation. A total of 40,000 images (8,000 from each class) were used for training. The remaining 2,000 images from each class were split equally and used for validation and testing. A similar setting was used for the other taxonomy as explained below. For the 4 class experiment, we have used 4 classes: normal, CIN1 (mild), CIN2 (moderate) and CIN3 (severe and carcinoma). For the three class classification, we followed the Bethesda system which is the standard format for reporting cervical cytology findings. The cell images were categorized into 3 classes: normal, mild as low- grade squamous intraepithelial lesion (LSIL), moderate, severe and carcinoma clubbed together as high grade squamous intraepithelial lesion (HSIL). Finally, for the 2-class classification, normal was taken as one class, and all the other four class were agglomerated into an abnormal class. This classification was performed to analyze the degree of separation among various classes, as elucidated by the accuracy of the model in separating them. Figure 4 shows the different taxonomies of cervical cell images employed on 3 different sets for cell image classification.

*Feature Extraction*

**Figure 5:**
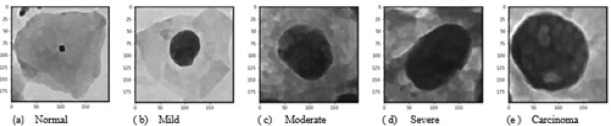
Original Enhanced Single Cell Cervical Images of All 5 Grades (Set 1)

**Figure 6:**
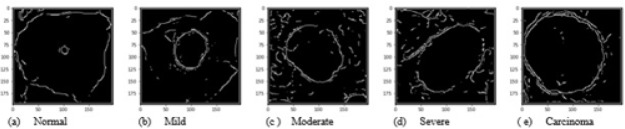
Contour Extracted Single Cell Cervical Images of All 5 Grades (Set 2)

**Figure 7:**
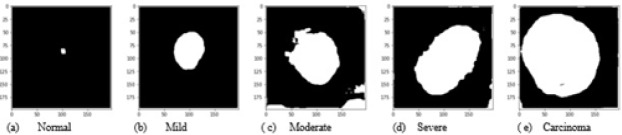
Binarized Single Cell Nucleus Portion of Cervical Images of All 5 Grades (Set 3)

Deep prediction model in convolution neural network can extract and predict the correct feature representations from the base features of images due to the number of trainable weights and biases in the network. The proposed CNN model with multiple processing layers aided in learning, different representations of data with multiple levels of abstraction to understand and extract essential features from cervical cell images for better image classification. We have considered three sets of images of normal, mild, moderate, severe and carcinoma for the study. To explain this hypothesis further, the examples of the various sets of images with their respective grades have been presented in Figures 5, 6 and 7. The first set, as shown in Figure 5 contains the original single cell images from Herlev dataset has achieved the accuracy of 94.1% in the 5 class case and the highest accuracies for most of the settings were obtained using the original enhanced images that includes all specific features (Mariarputham and Stephen, 2015) of nucleus and cytoplasm of cervical cell images.

Images were enhanced to improve the clarity by removing unwanted disturbances such as noise, poor contrast and blur contours using bi-histogram equalization with adaptive sigmoid function combined with sobel horizontal vertical mask. As the disease moves from benign to malignancy, variant changes occur in size, shape, texture, morphology and color of the nucleus and cytoplasmic features. The deep neural network architecture automatically learns the features of the cervical cells at multiple levels of abstractions to classify the cells to their respective grades. For the second set of images, as shown in Figure 6, we used canny edge detector to extract the edge information from the single cell images. The edge detection on cervical cell is essential as the shape and size of the nucleus and cytoplasm aids in the detection of the severity of the disease. For the third set of images as shown in Figure 7, we binarized the single cell Pap smear images of Herlev dataset, highlighting only the nucleus portion of cell images. The shape and size of the nucleus changes explicitly, as the severity of the disease progresses from normal, mild, moderate, severe and carcinoma. We used these three sets of images for the study to classify the cervical cell images as per the taxonomy shown in Figure 4 and evaluated and compared the performance of the model for all the classes in the three sets.

**Table 1:** Normal and Abnormal Category of Single Cell Cervical Images from Pap Smear Herlev Database

Normal	242 Cells
Superficial Squamous Epithelial Cells	74 cells
Intermediate Squamous Epithelial Cells	70 cells
Columnar Epithelial Cells	98 cells
Abnormal	675 Cells
Mild Squamous Dysplasia	182 cells
Moderate Squamous Dysplasia	146 cells
Severe Squamous Dysplasia	197 cells
Squamous cell carcinoma	150 cells

**Table 2.** Various Parameter Values Used for Augmentation of Cervical Single Cell Images from Herlev Dataset

Feature	Value
Rotation Range	180
Width Shift Range	0.2
Height Shift Range	0.2
Rescaling Factor	0.2
Shear Range	0.2
Zoom Range	0.3
Flip	Vertical and Horizontal

**Table 3:** Classification Accuracies of Cervical Single Cell Images on Various Datasets and Taxonomies

Data Used	5 class	4 class	3 class	2 class
Original Images	94.1	96.2	94.8	95.7
Contour Images	92.14	92.9	94.7	89.9
Binary Images	85.07	84.0	92.07	99.7

**Table 4:** Classification Accuracy of Single Cell Cervical Pap Smear Images Obtained from KMC (Kasturba Medical College), Manipal, Karnataka, India

Data Used	5 class	4 class	3 class	2 class
Original Images	95.31	94.62	94.8	96.11

**Figure 8:**
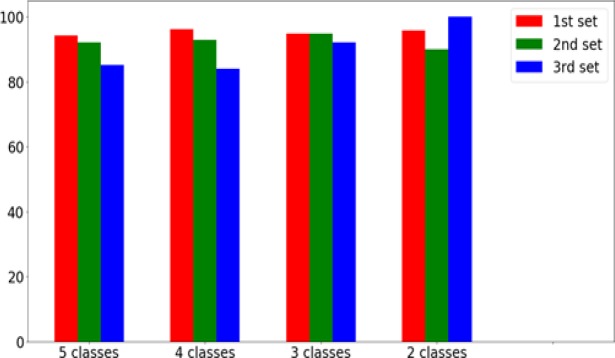
Comparison of Accuracies Obtained from the Proposed CNN Model for Different Classification Settings

**Figure 9:**
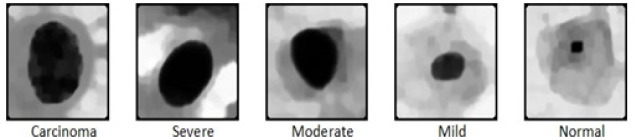
Single Cell Pap Smear Cervical Cell Images of All 5 Grades Obtained from Kasturba Medical College, Manipal, India

**Figure 10:**
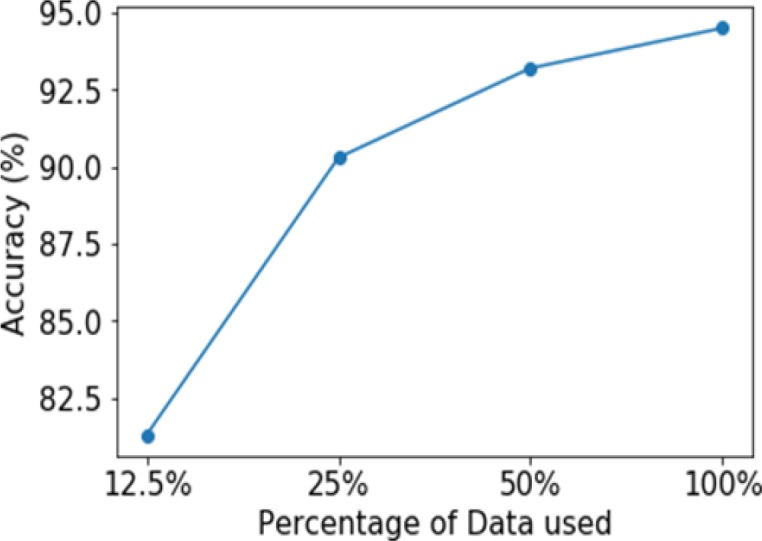
Graph of Accuracy Vs. Percentage of Data Used while Training the Model and Indicates How Accuracy is Effected by the Size of the Training Set Size

## Results

Using the proposed convolutional neural network model, the authors performed experiments on the different taxonomies of all the 3 sets of images. The results have been presented in Table 3.

The model has achieved the accuracy of 94.1% in the 5 class case. Similarly, the highest accuracies were obtained for most of the settings using the original enhanced images (set 1). It means that there are specific features in the images such as texture, size, shape and other essential features of cytoplasm and nucleus that aids the network to understand and learn the representation of images to emphasize for better classification.

However, as shown in Figure 8 accuracy obtained for the original single cell images (set 1) and contour images (set 2) are almost same with little variations among all the classes. In set 3 binarized images, as the class in- creases from 2 class to 5 class, the accuracy of the classification drastically decreases. This shows that, the accuracy of multi-level classification mainly depends on the different features present in the images. These features help the model to learn the image thoroughly for better multi-level classification. The accuracy for 2 class problems is the highest for the binarized image classifier, giving 99.7% of classification accuracy. This result can be attributed to the fact that since the size of the nucleus for the normal stage is much smaller than all the other stages, it can be used as a single differentiating factor for the normal vs. abnormal case. The other features introduce bias in the system and lead to spurious classifications as shown by the other classifier accuracies for the same case. It is also interesting to note that the accuracy obtained by the original image classifier (set 1) and contour image classifier (set 2) does not deviate much throughout the different settings, while those of the binarized image classifier changes the maximum through the different settings.

Inferring from these results, it can be understood that in the normal vs. abnormal case, the binarized images are extremely effective, and nucleus size can be used as the singular feature in classifying the images. However, as the number of class increases, the complexity of classification problem rises exponentially, and hence the model needs more features for improved accuracies. Finally, for the five class classification, the model needs all the features of the images for the best performance of the system. It is worth noting that across all the experiments, the architecture of the model is kept same.

The images which are obtained from Pathology department, Kasturba Medical College, Manipal, India were also tested with the proposed model. From the results inferred above, the proposed model needs all the features of the images to portray the best performance. So the original single image of set1 was used for the testing purpose. The tested images obtained from KMC (Kasturba Medical College), Manipal also showed good accuracy with the proposed CNN model shown in Table 4.

## Discussion

*Comparison with Previous Results*

We experimented our model with 3 sets of images. We considered the single cell enhanced images as the first set and achieved an accuracy of 94.1% for 5 class, 96.2% for 4 class, 94.8% for 3 class and 95.7% for 2 class problems. The second set includes contour extracted images which show 92.14%, 92.9%, 94.7% and 89.9% for 5, 4, 3 and 2 class problems, respectively. The third set of binarized images with the nucleus region of the cell shows 85.07% for 5 class, 84% for 4 class, 92.07% for 3 class and the highest accuracy of 99.97% for 2 class problems. When comparing all the 3 sets of images taken for the study, the complete single cell enhanced cell image shows good performance. The binarized shows poor performance for 5, 4 and 3 class problems but shows very good performance for 2 class problems. We compared our model with other approaches of the CNN model developed by different authors. Xu et al., (2016) worked on multimodal deep learning and used pre-trained AlexNet model for feature learning and achieved 88.91% of accuracy. Bora et al., (2016) also worked on AlexNet model and achieved 84 - 87% of accuracy before applying feature selection and 90 - 95% of accuracy after removing the redundant features. Zhang et al., (2017) worked on 2 class classification and achieved 98.3% accuracy and also performed classification for each of seven classes. Zhang et al., (2017) used AlexNet model as the feature extractor and coupled with a classic SVM classifier and achieved an accuracy of 99.19%. In our approach, we introduced our own CNN model and worked on 3 sets of images and checked the classification accuracy for 5, 4, 3 and 2 class problems. Upon comparing with the prior research published in cervical cell image classification, our model shows comparable, and in some cases, better results for different sets of images. For further study, our proposed model could serve as a template and be enhanced and refined with some ensemble for specific problems to achieve a higher classification accuracy.

*Ablation Study*

To study the effect of the training set size on the resultant accuracy of the model, the model was trained on differently sized subsets of the original image dataset (set 1). The results presented are only for multimodal classification, as comparable results were obtained for the rest of the sets. Figure 10 represents the importance of augmentation that aids in expanding the size of dataset. The accuracy decreases as the size of the dataset decreases, giving an accuracy of 90.3% with 25% of the original dataset and 95% with large volume of dataset. Thus, it can be observed that the augmentation of the dataset was an important reason for the efficient training of the model, resulting in a high accuracy.

In conclusion, this paper proposes a deep convolutional neural net- work based model to classify the cervical cell images for detecting malignancy. The enhanced images from Herlev dataset are augmented to increase the size of the dataset to improve the classification performance. The proposed deep learning model consists of three 2D convolution layers, each followed by activation and a max pooling layer. This model is trained with three sets of cellular images of Herlev dataset. The first set comprises original enhanced cervical cell images, the second set the contour extracted cell images, and the third set the nucleus highlighted binarized images. Several experiments were performed using different taxonomies for these images, to study the relative importance of features and the efficiency of the model. Our model performed well and achieved good performance with more number of training and testing sets. For the future work, the performance can be improved by using novel methods that aid in classifying the huge set of samples. On observing the classification accuracies of different models in different scenarios, it can be inferred that using an ensemble of such models should improve the results, and an ensemble will be able to remove the bias associated with a particular type of feature in the cellular images.
